# Clonal dissemination of methicillin sensitive and resistant Staphylococcus aureus among indigenous populations of the amazon and the southeast region in Brazil

**DOI:** 10.1186/2047-2994-4-S1-P194

**Published:** 2015-06-16

**Authors:** LM Abraão, CMCB Fortaleza, TA Barbosa, EP Lino Pereira-Franchi, DF Riboli, CH Camargo, RM de Souza, MDLR de Souza da Cunha

**Affiliations:** 1 Tropical Diseases, Botucatu Medical School, São Paulo State, Brazil; 2 Microbiology and Imunology, Biosciences Institute, Botucatu, Brazil; 3 Bacteriology, Adolfo Lutz Institute, São Paulo, Brazil; 4 Nursing, Federal University of Acre – UFAC, Cruzeiro do Sul, Brazil

## Introduction

Hospital-based studies have report high rates of *Staphylococcus aureus* and MRSA in indigenous populations. However, in Brazil there are no data regarded to native individuals *S. aureus* colonization or infection, mainly, regarded to *S. aureus* community dissemination.

## Objectives

Determine the molecular epidemiology of methicillin sensitive and resistant S. aureus from samples obtained from indigenous population in Brazil.

## Methods

328 samples (oral/nasal swabs) were taken of healthy indigenous villages located in Feijó and Mâncio Lima in Acre state, (Amazon region), and 115 were taken in Avaí, São Paulo State, (Southeast of the country). The samples were identified by traditional methods. Cefoxitin and oxacillin impregnated disks were used for antimicrobial susceptibility determination and PCR was employed for mecA detection and SCCmec characterization. Molecular typing was held by PFGE.

## Results

*S. aureus* carriage prevalence in Amazon region was 55.8% whereas in the southeast region was 59.1%. One nasal isolate was resistant to cefoxitin while 3 oral isolates were resistant to oxacillin; mecA gene was detected in 3 isolates, all of them SCCmec IV. MRSA prevalence in this study was 0.6% in Amazon region while MRSA was not detected in the southeast region. The *S. aureus* clonal analysis identified 12 clusters (>80% similarity) among native populations of both states; 7 of them with strains belonging to both regions studied. The cluster A, the biggest one with 25 isolates, grouped all the resistant strains to other sensitive isolates.

## Conclusion

We verified higher *S. aureus* prevalence compared to the prevalence in Brazilian non-natives (30%). MRSA was detected only among Amazon natives. The PFGE analysis showed that the resistant and sensitive strains likely have common origin. Thus, the dissemination of *S. aureus* with similar profiles among indigenous population living in extremely distant states in Brazil suggests a clonal dissemination of a possible peculiar *S. aureus* clone.

## Disclosure of interest

None declared.

